# Fatal Rodentborne Leptospirosis in Prison Inmates, South Africa, 2015

**DOI:** 10.3201/eid2605.191132

**Published:** 2020-05

**Authors:** Kovashnee Naidoo, Mark Moseley, Kerrigan McCarthy, Ruvimbo Chingonzoh, Charlene Lawrence, Grace M. Setshedi, John Frean, Jennifer Rossouw

**Affiliations:** National Institute for Communicable Diseases, a division of the National Health Laboratory Service, Johannesburg, South Africa (K. Naidoo, K. McCarthy, R. Chingonzoh, G.M. Setshedi, J. Frean, J. Rossouw);; University of Aberdeen, Aberdeen, Scotland, UK (M. Moseley);; Western Cape Government: Health, Cape Town, South Africa (C. Lawrence);; University of the Witwatersrand, Johannesburg (J. Frean)

**Keywords:** Zoonoses, invasive hosts, molecular epidemiology, public health, outbreak, prison, South Africa, Rattus norvegicus, bacteria, rats, leptospirosis, Leptospira

## Abstract

Leptospirosis is a neglected zoonotic disease. In 2015, leptospirosis was diagnosed in 2 prison inmates in South Africa. Using real-time PCR and DNA sequencing, we identified *Leptospira interrogans* serogroup Icterohaemorrhagiae in rodents and water samples within the prison. Leptospirosis might be frequently underdiagnosed in South Africa.

Although leptospirosis, a bacterial zoonosis, is responsible for ≈1 million cases per year worldwide, estimates of its incidence in Africa are limited by a lack of quality-assured studies (*1*). Humans become infected through mucosal membranes or skin breaks by direct contact with reservoir animals or exposure to urine-contaminated soil or water. We describe an outbreak of leptospirosis in prison inmates in Cape Town, South Africa, and identification of probable animal sources and environmental routes of infection.

In September 2015, the South Africa Department of Correctional Services requested the National Institute for Communicable Diseases to assist with investigation and management of leptospirosis infections in 2 inmates at a maximum-security prison in Cape Town. The National Health Laboratory Service Animal Ethics Committee clearance 131/11 granted approval for rodent trapping and testing; ethical clearance certificate no. M160667 from the Human Research Ethics Committee (Medical) of the University of the Witwatersrand covered the outbreak investigation.

Case-patient 1, a 52-year-old man, was admitted to a hospital in Cape Town. He had jaundice, overwhelming sepsis, disseminated intravascular coagulation, and multiorgan failure after ≈1 week of worsening illness that included conjunctivitis, myalgia, and fever. At admission, his leukocyte count was 18.64 × 10^9^ cells/L (reference 4–10 × 10^9^ cells/L), platelets 65 × 10^9^/L (reference 137–373 × 10^9^/L), total bilirubin 30.23 mg/dL (reference 0–1.23 mg/dL) (91% conjugated), alanine transaminase 95 U/L (reference 5–40 U/L), and creatinine 10 mg/dL (reference 0.72–1.18 mg/dL). He died the following day.

Case-patient 2, a 49-year-old man occupying the same prison cell as case-patient 1, was hospitalized 10 days later. He had jaundice, abdominal pain, anorexia, weakness, and body aches. The initial leukocyte count was 21.71 × 10^9^ cells/L, platelets 100 × 10^9^/L, total bilirubin 17.89 mg/dL (88% conjugated), alanine transaminase 66 U/L, and creatinine 4.66 mg/dL. The patient received treatment and was discharged after 9 days of hospital stay.

A physician diagnosed leptospirosis on the basis of clinical signs and clinical pathology consistent with severe leptospirosis (leukocytosis, thrombocytopenia, hyperbilirubinemia, mild transaminasemia, acute renal failure). For both patients, blood cultures and hepatitis virus tests were negative. However, IgM to *Leptospira* spp. was detected by the Panbio *Leptospira* IgM ELISA (Standard Diagnostics, https://www.alere.com), confirming the clinical diagnosis. The reference serologic method (microagglutination test) is not available in South Africa, and neither blood nor urine samples were available for subsequent analysis by the reference laboratory.

An initial assessment of the prison by the National Institute for Communicable Diseases outbreak investigation team, together with provincial and national Department of Health officials, identified conditions favorable for *Leptospira* transmission (rodent infestation, standing water, and food waste accumulation). Within 3 weeks after hospital admission of case-patient 2, 12 *Rattus norvegicus* rats, identified by morphologic characteristics, were live-trapped around the prison section housing the patients and humanely euthanized, and their kidneys were harvested. Five stagnant drain water samples were collected in the same area.

We extracted DNA from rodent kidney samples using the QIAamp DNA Mini kit on a QIAcube system (QIAGEN, https://www.qiagen.com) and from water samples using ZR Soil Microbe DNA MicroPrep kit (Zymo Research, https://www.zymoresearch.com). We detected *Leptospira* DNA with an Applied Biosystems 7500 qPCR instrument (Applied Biosystems, https://www.thermofisher.com) using published primers targeting ≈300 bp of the *lfb1* gene (*2*) and sequenced the amplicons as previously described (*3*). *Leptospira lfb1* sequences are available in GenBank under accession nos. MH795484–MH795490 (rodents) and MH795482–MH795483 (water).

The prevalence of *Leptospira* infection in rodents was 66.7% (8/12), and we detected *Leptospira* DNA in 40% (2/5) of water samples. Sequencing of the *lfb1* amplicons from both rodent and water samples identified a single genotype. Phylogenetic analysis using MEGA7 (*4*) revealed 100% identity to strains isolated from humans in New Caledonia and typed as *L. interrogans* serogroup Icterohaemorrhagiae (Figure) (*5*), a serogroup identified in historical cases from Cape Town (*6*,*7*).

**Figure:**
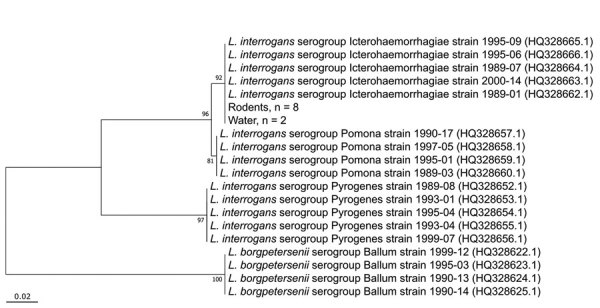
Neighbor-joining phylogenetic tree based on 256 bp of *Leptospira lfb1* gene of isolates from South Africa and reference sequences. *Leptospira* sequences collected during this study are labeled according to sample source (rodent, water) and the number of sequences indicated. Reference sequences include *lfb1* sequences from isolates obtained from human cases of leptospirosis in New Caledonia (*3*). GenBank accession numbers are provided in parentheses. Scale bar indicates nucleotide substitutions per site.

*L. interrogans* serogroup Icterohaemorrhagiae, traditionally associated with rats, is frequently implicated in fatal leptospirosis and survives for prolonged periods in fresh water (*8*). Therefore, rodent infestations, the high prevalence of infected rodents, and increased environmental contamination are likely to have contributed to this prison outbreak of human leptospirosis. To reduce inmate exposure, the affected section of the prison was evacuated before intensive clean-up and rodent control activities were undertaken. Moreover, early referral of inmates with nonspecific febrile illness for preemptive treatment of leptospirosis was implemented. Thirty serum samples from symptomatic inmates were referred for *Leptospira* spp. IgM ELISA and real-time PCR; all tested negative, possibly because of the intermittent nature of leptospiraemia and the lack of detectable antibodies during early stages of the disease (*9*). No additional cases of leptospirosis were diagnosed.

We found evidence that rodent infestation and contaminated environments within confined settings, such as prisons, are risk factors for leptospirosis in humans, supporting previous findings from an Ecuador prison (*10*). Moreover, the finding that highly pathogenic *Leptospira* spp (*6*,*7*). continue to circulate in rodents suggests that human leptospirosis may be an underreported public health problem in South Africa, particularly among persons living in informal settlements, where rodent infestations are common and environmental conditions favor disease transmission.
